# Genetically confirmed chronic granulomatous disease in a Kenyan child: case report

**DOI:** 10.3389/fimmu.2023.1172848

**Published:** 2023-05-18

**Authors:** Diana Marangu-Boore, Fred Kambuni, Mary Onyinkwa, Stalin Ramprakash, Raghuram C.P., Brian Eley, Sagar Bhattad

**Affiliations:** ^1^ Paediatric Pulmonology Division, Department of Paediatrics and Child Health, University of Nairobi, Nairobi, Kenya; ^2^ Paediatric Surgery Division, The Nairobi Hospital, Nairobi, Kenya; ^3^ Radiology Department, The Nairobi Hospital, Nairobi, Kenya; ^4^ Pediatric Hemat-oncology and Bone Marrow Transplant (BMT), Department of Pediatrics, Aster CMI Hospital, Bangalore, India; ^5^ Paediatric Infectious Diseases Unit, Department of Paediatrics and Child Health, Red Cross War Memorial Children’s Hospital, University of Cape Town, Cape Town, South Africa; ^6^ Pediatric Immunology and Rheumatology Division, Department of Pediatrics, Aster CMI Hospital, Bangalore, India

**Keywords:** Africa, inborn errors of immunity, mycobacteria, primary immunodeficiency, tuberculosis

## Abstract

**Introduction:**

We report the first case of genetically confirmed chronic granulomatous disease (CGD) in a Kenyan child.

**Clinical findings:**

A 7-month-old male infant, the only child of non-consanguineous parents, presented with cough, fever, fast breathing, oral thrush, and axillary lymphadenopathy ipsilateral to the Calmette–Guérin bacillus scar. He had been hospitalized 5 weeks prior for severe pneumonia. Plain chest radiography showed bilateral patchy airspace opacification; chest computed tomography revealed multiple large lung nodules and left axillary lymphadenopathy. HIV ELISA was negative; tuberculin skin test was positive; lymph node biopsy macroscopically revealed caseous granulomas seen on histology; isoniazid- and rifampicin-susceptible *Mycobacterium tuberculosis* complex isolate was detected on the Hain test. First-line anti-tuberculous drugs were added to his empiric treatment comprising piperacillin–tazobactam, amikacin, cotrimoxazole, and fluconazole. He was discharged after 10 days based on clinical resolution.

**Diagnoses, interventions, and outcome:**

An inborn error of immunity (IEI) was considered given the recurrent fevers and atypical lung nodules. Genetic analysis revealed a hemizygous pathogenic variant on *CYBB* in keeping with X-linked CGD. The child’s fevers recurred 2 weeks post-discharge but completely resolved on prophylactic itraconazole and cotrimoxazole. He underwent a successful haplo-identical hematopoietic stem cell transplantation at an experienced center in India with his father as the donor and is currently doing well on post-transplant follow-up.

**Conclusion:**

Genetic testing is relatively accessible and cost-effective for the diagnosis of IEI in low-and-middle-income countries. Expert multi-disciplinary collaboration is key for successful outcomes.

## Introduction

Chronic granulomatous disease (CGD) is an inborn error of immunity affecting the function of phagocytes resulting from attenuation or absence of NADPH oxidase activity (1). Genetically, CGD and CGD-like disease are heterogeneous diseases with an X-linked recessive form caused by mutations in *CYBB* gene (2) and an autosomal recessive form caused by mutations in the *CYBA*, *CYBC1* (3), *NCF1*, *NCF2*, *NCF4*, and *RAC1/2* genes (4, 5). In Europe, the USA, and Latin America, the X-linked form of CGD is the most common (6–8). The majority of African studies on inborn errors of immunity (IEIs) are from Northern African countries including Algeria, Egypt, Libya, Morocco, Sudan, and Tunisia (9, 10), where consanguineous marriage is highly prevalent; thus, most diseases tend to be autosomal recessive (9). Data on CGD in East Africa are lacking. We report on the first case of X-linked CGD with a hemizygous pathogenic variant in *CYBB* gene in a Kenyan child.

## Case description

**Figure 1:**
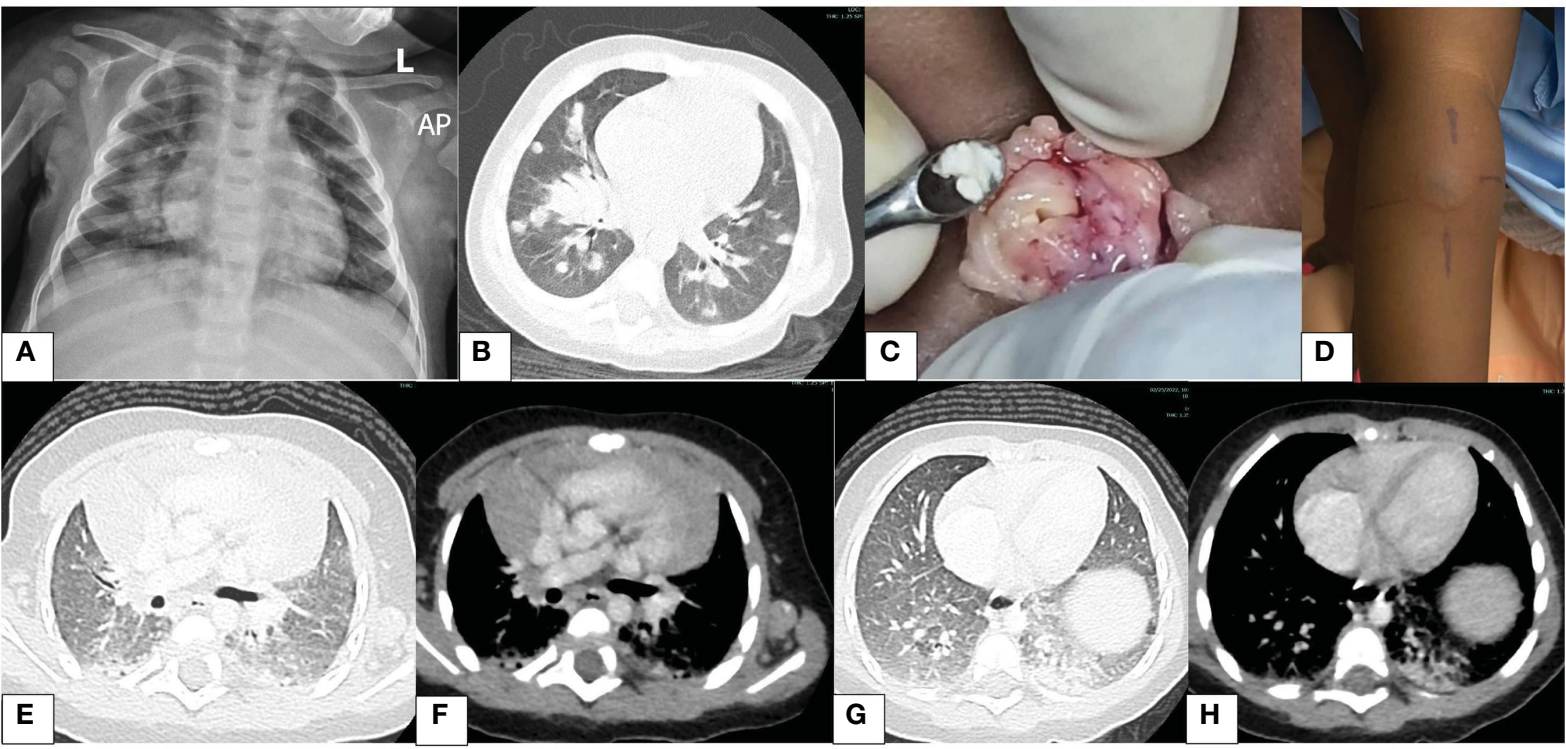
Images illustrating findings of pertinent investigations. **(A)** Chest X-ray: right lower zone and left retrocardiac opacification (day 1). **(B)** Chest CT: lung window—multiple nodules (day 2). **(C)** Intraoperative findings of the left axillary node; note the caseous material (day 4). **(D)** Tuberculin skin test at 72 h shows a large induration; the inner edges of the lines drawn depict the outer edge of the induration; this measured 26 mm (day 5). **(E)** Chest CT: lung window—nodule resolution (month 4). **(F)** Chest CT: mediastinal window—thymic rebound (month 4). **(G)** Chest CT: lung window—bibasal pneumonia, left > right (month 4). **(H)** Chest CT: mediastinal window—bibasal pneumonia (month 4).

A 7-month-old male infant who had been unwell for 5 weeks prior to hospitalization presented with persistent fevers, cough, and fast breathing despite being on treatment for pneumonia with broad-spectrum antimicrobials comprising crystalline penicillin with gentamicin, ceftriaxone, and meropenem at a different health facility. Pertinent in family history, he was the only child of non-consanguineous parents. On examination, the child appeared sickly, was febrile, and had oral thrush and left axillary lymphadenopathy ipsilateral to the Calmette–Guérin bacillus (BCG) scar. He was tachypneic, had mild lower chest wall indrawing, and had normal oxygen saturation in room air. Good air entry was heard on auscultation bilaterally with few basal crepitations bilaterally. He was evaluated for the current illness and the possible underlying IEI. Details of investigations have been tabulated (**Table 1**). Plain chest radiography at admission showed bilateral airspace opacities (**Figure 1A**). We commenced piperacillin–tazobactam, amikacin, high-dose cotrimoxazole, and fluconazole for the persistent pneumonia and candidiasis. Chest computed tomography (CT) revealed multiple bilateral large lung nodules (**Figure 1B**). Left axillary lymph node biopsy macroscopically revealed caseous material (**Figure 1C**). Lymph node tissue histology revealed granulomas suggestive of mycobacterial infection, but no acid-fast bacilli were seen. Periodic-acid Schiff stain for fungi was negative. The tuberculin skin test was positive, measuring 26 mm at 72 h (**Figure 1D**). First-line anti-tubercular therapy comprising isoniazid, rifampicin, pyrazinamide, and ethambutol was initiated.

**Table 1:** Summary of investigations.

Test	Results
Full blood count	Leukocytosis: 20.6 × 10^9^/L Neutrophilia: 9.4 × 10^9^/L Monocytosis: 2.8 × 10^9^/L Normal lymphocyte count: 8.3 × 10^9^/L Red blood cells: 4.45 × 10^12^/L Hemoglobin level: 8.3 g/dl Mean corpuscular volume (microcytic): 61.3 fl Mean corpuscular (hypochromic): 18.7 pg Mild thrombocytosis: 484 × 10^9^/L
HIV ELISA	Negative
Flexible bronchoscopy	Unremarkable
Bronchoalveolar lavage (BAL)	Cytology: suggestive of chronic inflammation BioFire Respiratory 2.1 panel including SARS-CoV-2: negative Xpert MTB/RIF: negative Bacterial culture: negative Fungal culture: negative Mycobacterial culture: negative
Antinuclear antibody	Negative
Rheumatoid factor	Negative
Immunoglobulin G, A, and M concentrations	Normal
T, B, and NK lymphocyte subset concentrations	Normal

## Diagnoses, interventions, and outcome

BCG disease was a differential diagnosis based on axillary lymphadenopathy on the arm with the BCG scar. We were cognizant that BCG would be resistant to pyrazinamide. Fevers nonetheless subsided 72 h after beginning anti-tubercular therapy, and we chose a clinical approach pending further mycobacterial test results. We also considered CGD as a possible differential diagnosis because the infant was HIV negative and had recurrent fevers, probable BCG disease, and lung nodules on the CT chest that were not typical of tuberculosis (TB). Nitroblue tetrazolium (NBT) reduction and dihydrorhodamine reduction (DHR) tests to demonstrate an absent respiratory burst were not available in the country and could not be outsourced. Notably, lymphocyte subset testing is outsourced overseas for patients outside HIV programs.

On day 7 of admission, we sent blood *via* courier for genetic testing to rule out CGD or other IEI to INVITAE, a genetic laboratory in the USA. The targeted primary immunodeficiency panel employed next-generation sequencing and tested 407 genes including genes implicated in CGD. Permission was sought from this lab to use the genetic sequencing data for publication. On day 9 of admission, bronchoalveolar lavage (BAL) bacterial and fungal culture results returned negative. On day 10 of hospitalization, the Genotype MTBDRplus VER 2.0 (Hain) test result of lymph node tissue was received, detecting a *Mycobacterium tuberculosis* complex isolate susceptible to isoniazid and rifampicin. Following 10 days of empiric intravenous antimicrobials, the boy was discharged in a clinically stable condition. The plan was to complete 14 days of fluconazole and 21 days of therapeutic cotrimoxazole and continue anti-mycobacterial treatment as an outpatient.

Two weeks following hospital discharge, he developed new fevers. Prophylaxis with cotrimoxazole and itraconazole was started with clinical improvement. Anti-TB medication was continued. After 2 months of TB therapy, the follow-up CT chest showed complete resolution of the lung nodules; however, bilateral basal ground glass opacification was noted (**Figures 1E–H**). Notably during this time, itraconazole had run out, and the caregiver interchanged it with fluconazole. We educated the parents on the importance of compliance with the prescribed regimen. The mycobacterial culture was negative on bronchoalveolar lavage; results from lymph node biopsy were not found. The child completed 6 months of first-line TB treatment and was clinically well. Later, levofloxacin was added to cover for BCG disease.

We received the primary immunodeficiency genetic panel results 3 weeks post-discharge, which revealed a pathogenic hemizygous non-sense variant (c.736C>T, p.Gln246*) in exon 7 of *CYBB* gene in keeping with X-linked CGD. With these results, the team in Kenya consulted IEI experts in South Africa and India, who recommended hematopoietic stem cell transplantation (HSCT) to achieve a cure. We counseled his parents and extended family on his condition, all of whom were very receptive and keen on the HSCT. Both biological parents were screened for the *CYBB* mutation, and his mother was found to be a heterozygous carrier for the same variant.

He underwent a successful haplo-identical HSCT at the age of 1 year 2 months at an experienced center in India with his father as the donor (5/10 HLA match). The conditioning regimen consisted of anti-thymocyte globulin 7.5 mg/kg on day −11 to −9, fludarabine 180 mg/m^2^ on day −9 to −4, IV busulfan on day −7 to −4 (targeted AUC 86.4 mg/L), and thiotepa 10 mg/kg on day −3. The source of the stem cells was granulocyte colony-stimulating factor (G-CSF)-primed unmanipulated bone marrow. Post-transplant cyclophosphamide (50 mg/kg on days +3 and +4), tacrolimus, and mycophenolate mofetil were used for graft-versus-host disease prophylaxis. The post-transplant neutrophil engraftment was achieved on day 15 and platelet engraftment on day +18. His last chimerism was 100% donor, and his immunosuppression was stopped on day +273. Major complications experienced were subclinical cytomegalovirus (CMV) reactivation and prolonged norovirus infection, which both resolved with treatment. The child is currently doing well more than 10 months post-transplant with no current infections or any evidence of graft-versus-host disease.

## Timeline

**Figure 2:**
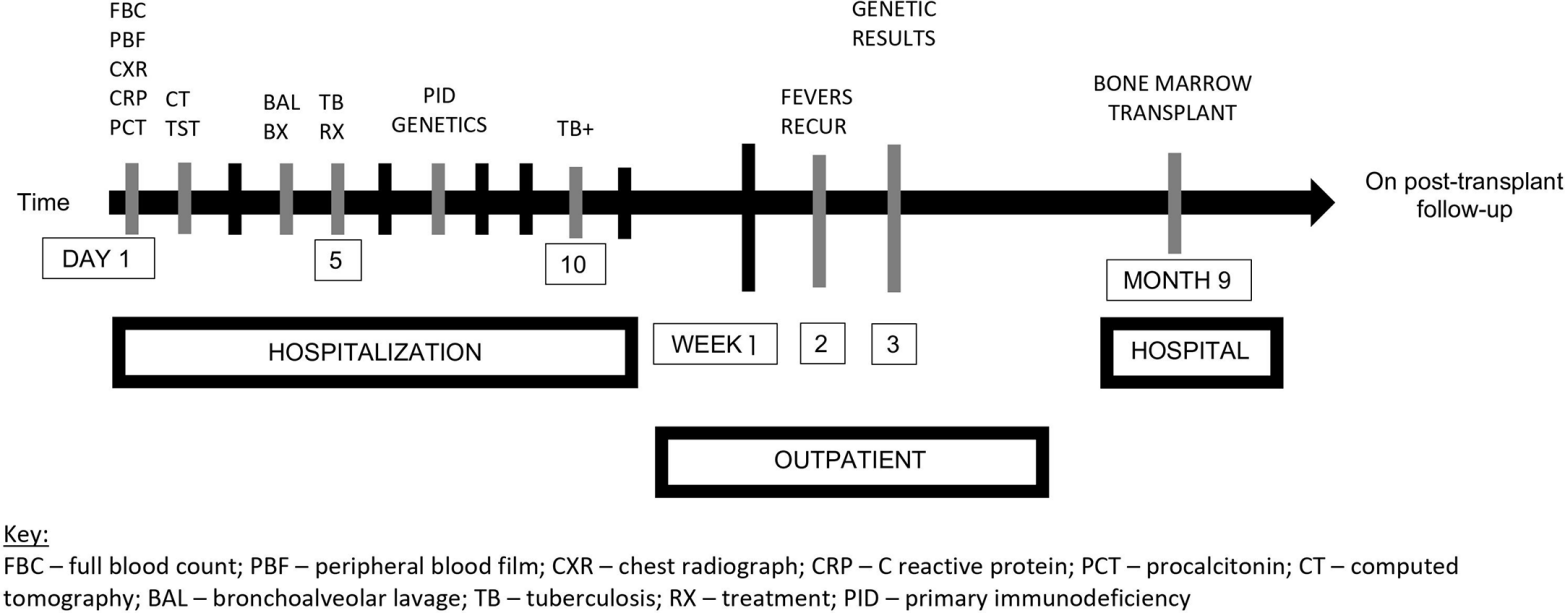
Timeline of events. FBC, full blood count; PBF, peripheral blood film; CXR, chest radiograph; CRP, C reactive protein; PCT, procalcitonin; CT, computed tomography; BAL, bronchoalveolar lavage; TB, tuberculosis; RX, treatment; PID, primary immunodeficiency; +, positive.

A timeline of events is illustrated in **Figure 2**.

## Discussion

To our knowledge, this is the first genetically confirmed case of X-linked CGD in Kenya. The infant had a hemizygous pathogenic variant on *CYBB* that has been submitted to the National Center for Biotechnology Information (11). This variant has been documented from cohorts in China, France, the USA, and Turkey (6, 12–15) but is not in population databases. Interestingly, the first genetically confirmed case of CGD in South Africa published over 10 years ago had a novel *CYBB* mutation (16). A retrospective analysis conducted recently in the same South African center included two more CGD cases: one autosomal recessive (AR) and the other with an undefined mutation (17). In Western countries, 65%–70% of CGD cases are X-linked (18, 19). Conversely, in a cohort of 15 children with CGD in Egypt, the most common gene mutation was CYBA (10). The targeted gene panel used in this study does not detect the hotspot deletion in *NCF1* (20), which might be the most common variant in the Middle East (21), because there is a pseudogene that gets sequenced instead of *NCF1* (20). The true burden of CGD and other IEI in low-and-middle-income countries is unknown, particularly in East Africa. In Kenya, only one case of DiGeorge syndrome (22) and three cases of severe combined immune deficiency (23) have been documented in the literature. More data on CGD and other IEI are needed in the Kenyan context to inform the health system and improve patient care.

The first step to addressing the diagnostic challenge for CGD in low-and-middle-income countries lies in increasing the clinician’s level of suspicion. Our patient presented early in infancy with recurrent infections, which is typical in children with X-linked CGD as compared to those with AR-CGD. Children with CGD are susceptible to mycobacterial infections. These include TB and complications from the BCG vaccine such as scarring or abscess formation at the BCG injection site and disseminated BCG infection since the vaccine is a live attenuated form of *Mycobacterium bovis* (24). We suspected BCG disease in this child due to the clinical presentation: axillary lymphadenopathy on the same side as the BCG scar, the strongly positive tuberculin skin test, and multiple large lung nodules that could be pulmonary granulomas (25). Although the Hain test on lymph node biopsy detected *M. tuberculosis*, which was sensitive to both isoniazid and rifampicin, this did not rule out *M. bovis*, which is also part of the *M. tuberculosis* complex. Results of the mycobacterial culture of the lymph node biopsy were not found. Interestingly, Xpert MTB/RIF and mycobacterial culture in bronchoalveolar lavage were negative, underscoring the need to sample different specimens to improve the yield for a bacteriological diagnosis of TB in children (26). Our patient’s diagnosis of a mycobacterial infection highlights the double burden of infectious diseases and non-communicable diseases particularly in low-and-middle-income countries. Kenya is among the top 30 high TB burden countries in the world (27). Furthermore, patients with CGD are also susceptible to catalase-positive organisms such as *Staphylococcus aureus*, *Burkholderia cepacia* complex, *Serratia marcescens*, *Nocardia* species, and *Aspergillus* species in the lungs, lymph nodes, skin, liver, and bones (19). None of these infections were confirmed in our case despite extensive testing.

Second, diagnostic testing for CGD needs to be made available and affordable. Presently, NBT and DHR tests are not available in Kenya and were not performed in our patient. DHR testing remains an important first-line screening tool for CGD. This functional assay in comparison to NBT is easier to perform, more robust, more quantitative, and more sensitive (28) and helps in identifying carriers in X-linked CGD (29). It is worth noting that these tests have some drawbacks. Mondal et al. have illustrated how CGD can be missed if NBT alone is performed in patients with residual NADPH activity, despite having typical clinical signs (30). Furthermore, performing DHR with different stimulants may help clinch the diagnosis, particularly using milder neutrophil stimulants such as *Escherichia coli* or *S. aureus* for CGD with residual enzyme activity (30). Corroborating NADPH enzyme activity and the genetic results would have been important for diagnostic confirmation especially if the patient had a novel mutation. For CGD and other IEI, genetic testing is key in that it guides management, enables access to specific treatments, and facilitates genetic counseling. Although cases are increasingly being documented, one of the impediments to genetic diagnosis cited is limited finances (31). At the time, INVITAE was charging 250 US dollars (presently 350 US dollars) for the targeted panel excluding courier charges. This may be relatively affordable to obtain a definitive diagnosis in patients suspected to have IEI. This cost includes testing for additional diseases in the same clinical domain, such as primary ciliary dyskinesia, and cystic fibrosis, as well as family testing in some cases if the patient is found to have a pathogenic mutation. Furthermore, sending the sample to the USA *via* courier leveraged existing logistic and transport systems, making this testing relatively available for patients in Africa (32). Engelbrecht et al. reported on a South African experience utilizing whole-exome sequencing and targeted panels to identify IEI in children with a history of severe, unusual, persistent, and/or recurrent TB or was suspected to have Mendelian susceptibility to mycobacterial disease. Through this next-generation sequencing technique, they achieved a molecular diagnosis in 30% of the patients (33). Recently, the Asian Primary Immunodeficiency Network proposed targeted gene Sanger sequencing as the first-tier genetic test for children suspected to have the five common X-linked IEI: X-linked agammaglobulinemia, Wiskott–Aldrich syndrome, X-linked CGD, X-linked severe combined immunodeficiency, and X-linked hyper-IgM syndrome (34). The optimal approach to the choice of genetic testing for CGD and other IEI will need to be contextualized. Advocating for genetic testing to be catered by governments, provided, for example, by the National Health Insurance Fund in Kenya as part of Universal Health Coverage, would be strategic and sustainable (35).

Last but not least, expert multi-disciplinary collaboration is essential for optimal patient outcomes. Our patient’s results revealed a non-sense variant (c.736C>T, p.Gln246*) in exon 7 of *CYBB*, which we shared with expert immunologists in South Africa and India. Following discussions, we commenced the infant on cotrimoxazole and itraconazole prophylaxis and planned for HSCT at an experienced center, as has been documented in the literature (18, 19, 36). Presently, HSCT is the only curative option. Indications that have been published for HSCT in patients with CGD vary widely, from transplant for all patients if a matched donor can be identified, to transplant if a matched donor is identified and there is no residual oxidase activity or severe disease complications develop. The indications stipulated by the European Society for Immunodeficiencies and the European Blood and Marrow Transplantation have been found to be more specific than most recommendations and are eventually met by most patients with CGD (37). In the USA, allogeneic HSCT for CGD has been shown to be associated with reduced incidence of infection and improved functional performance but not with a change in overall survival. Transplant at age ≤ 14 years is associated with improved transplant-related survival (93% *vs.* 82% at 5 years post-transplant) (38). The survival for CGD following HSCT in European patients is 88% at a median follow-up of 2.3 years, significantly better at 96% ± 4% for children transplanted at age ≤ 8 years and 100% for children without active complications at HSCT (39). In the absence of matched family or unrelated donors, a haploidentical transplant offers an opportunity for a cure in these children. A review of nine studies on haploidentical transplants for CGD comprising 24 participants, 20 of whom were children aged 44 months to 16 years, revealed a chimerism of 90%–100% on the last follow-up. Haploidentical transplantation in a 3-year-old child with autosomal recessive CGD due to *NCF1* gene mutation has been previously employed successfully at this center in India. Bhattad et al. report their approach to be effective, affordable, and undertaken without delays even in limited resource settings (40). Further research to optimize other modalities for CGD treatment including gene therapy and reactive oxygen species-producing enzyme replacement therapy may need to be explored in developing countries (41, 42).

Notwithstanding the gaps highlighted, we acknowledge the patient’s family’s resilience and the clinical team’s continuous concerted efforts to improve the care we provided. This case has been pivotal for our institutions’ learning and continuous improvement in identifying and managing children with CGD and other IEI.

## Data availability statement

The original contributions presented in the study are publicly available. This data can be found here: https://doi.org/10.6084/m9.figshare.22561513.

## Ethics statement

Ethical review and approval was not required for the study on human participants in accordance with the local legislation and institutional requirements. Written informed consent to participate in this study was provided by the participants’ legal guardian/next of kin.

## Author contributions

DM-B drafted the initial manuscript with support from BE and SB. All authors contributed to the data that form the basis of this manuscript. All authors contributed to the article and approved the submitted version.
